# Additive Manufacturing of Thermochromic Chitosan Hydrogels for Battery-Free Colorimetric Thermal Indication

**DOI:** 10.3390/polym18192319

**Published:** 2026-09-22

**Authors:** Jaewon Lim, Na Kyoung Kim, Jong Woo Hah, Yoo Sei Park, Inhwan Lee, Hyoryung Nam, Geon Hwee Kim

**Affiliations:** 1 Department of Mechanical Engineering, Chungbuk National University (CBNU), 1, Chungdae-ro, Seowon-gu, Cheongju 28644, Republic of Korea; 47899@naver.com (J.L.); jussy1822@gmail.com (N.K.K.); hajw2001@gmail.com (J.W.H.); anxanx@chungbuk.ac.kr (I.L.); 2 Department of Nanoenergy Engineering, Pusan National University, Busandaehak-ro 63 Beon-gil 2, Geumjeong-gu, Busan 46241, Republic of Korea; yoosei@pusan.ac.kr; 3 Department of Nano Fusion Technology, Pusan National University, Busandaehak-ro 63 Beon-gil 2, Geumjeong-gu, Busan 46241, Republic of Korea; 4 Department of Biomedical Engineering, Daegu Catholic University School of Medicine, 33 Duryugongwon-ro 17-gil, Nam-gu, Daegu 42472, Republic of Korea

**Keywords:** thermochromic hydrogel, colorimetric thermal indication, passive thermal indication, multilayer hydrogel, direct ink writing

## Abstract

Battery-free, at-a-glance thermal readouts are attractive for point-of-care triage and low-resource settings. However, three-dimensional thermochromic hydrogel printing remains challenging because delayed gelation can compromise linewidth control and multilayer structural fidelity. In this study, a syringe-pump-driven extrusion-based direct ink writing platform was integrated with a temperature-controlled coagulation bath to induce immediate in situ gelation during thermochromic chitosan hydrogel printing. Systematic tuning of the nozzle gauge, printing speed, and flow rate, combined with rapid thermal gelation in the heated coagulation bath, mitigated die swelling, with the die-swelling ratio, which ranged from 2.1 to 3.7 at low printing speed, decreasing to approximately 1.0 at intermediate speeds. This enabled parameter-dependent control of strand diameter and reproducible multilayer fabrication. The mechanical properties of the printed hydrogels were further tuned by varying the coagulation bath temperature and chitosan concentration; gelation at 60 °C yielded the highest ultimate tensile strength (0.172 MPa), and a 4.0 wt% chitosan formulation provided the most balanced combination of strength and extensibility. Adjusting the thermochromic pigment composition modulated chromatic separation across temperature states in CIE L*a*b* color space over 26–54 °C. Formulation tuning broadened the separation of color states within an intermediate range of 31–38 °C, which spans normal skin temperature and the febrile range relevant to fever detection. The colorimetric response remained stable over 50 heating–cooling cycles, with color differences below the threshold of visual perceptibility. A multilayer construct further demonstrated layer-resolved colorimetric readout within a single 3D structure, indicating the potential of this passive hydrogel indicator for battery-free, spatially resolved thermal indication in this temperature range.

## 1. Introduction

Skin-surface temperature is a fundamental physiological variable and a sensitive proxy for localized changes in the thermal state of the body, motivating continuous monitoring in wearable formats for applications ranging from body temperature tracking to thermal-alarm scenarios [1,2,3]. Recent advances in flexible sensors indicate a growing expectation that soft, skin-interfaced platforms should support reliable operation under bending and stretching because the skin surface is curvilinear, mechanically deformable, and subjected to deformation during day-to-day motions [1,4,5]. Practical deployment further requires comfort-oriented attributes such as lightweight form factors, conformal contact, and architectures that remain functional without bulky packaging, so that temperature information can be captured and interpreted in real-world settings rather than only under benchtop conditions [2,4,6,7].

Despite these requirements, many wearable temperature sensors continue to adopt electrical transduction, such as resistive- or impedance-based readouts using conductive hydrogels or ionogels [1,2,6]. Although these platforms can provide quantitative signals, they still require electronics for signal acquisition and careful integration of electrodes, interconnects, and readout hardware [1,6,8,9]. In practice, many flexible temperature-sensing systems based on electrical readouts still require integration with electrical transmission modules, data acquisition units, power supplies, and display interfaces, highlighting the nontrivial packaging and interfacing steps that can accompany electrical readouts even when the sensing layer itself is soft and stretchable [6,8,10]. These integration requirements can complicate low-profile, disposable implementations and may not align with point-of-care or field situations in which immediate, human-readable interpretation is preferred for rapid triage [1,7,11]. Collectively, these considerations motivate the development of a complementary class of passive thermal indicators that directly translate temperature-dependent material changes into visually interpretable information without requiring electrical power, signal-conditioning circuitry, or dedicated readout electronics [9,12,13,14,15].

Thermochromic materials offer a direct path to this paradigm by converting temperature into an optical response that can be read instantly, which is particularly attractive for rapid triage and thermal-alarm use cases in point-of-care or resource-limited settings where immediate, shared interpretation matters more than continuous numerical logging [3,9,12,13,16,17]. Because the temperature-dependent optical response can be interpreted visually, such materials enable passive thermal indication without requiring an external power supply or electronic signal-processing components. In this context, microencapsulation is widely used to incorporate thermochromic components into inks and films while preserving visual functionality [12,18,19]. However, simple visual functionality alone is insufficient for the practical use of thermochromic systems. Across thermochromic applications, key constraints governing practical utility include how accurately temperature-dependent color transitions can be calibrated and clearly distinguished, and how well the materials are matched to the intended fabrication process and device format. Limited color contrast can hinder the discrimination of different thermal states, whereas poor fabrication and device compatibility can impede practical implementation [12,20,21].

These constraints become more difficult to address when visual readout is expected to be spatially structured rather than planar. Additive manufacturing provides an attractive route for introducing geometric complexity and spatially programmed functionality into such responsive materials [3,18,22]. Recent studies have demonstrated direct-ink-written thermochromic composites with programmable temperature-dependent color and deformation [18], and extrusion-printed thermochromic lattice membranes have been applied to skin-attachable fever detection [3]. In parallel, thermo-responsive hydrogels whose optical properties switch near body temperature have been developed for skin-mountable sensing [23]. More broadly, 3D printing has been increasingly applied to hydrogel-based flexible sensors because it enables customizable geometries and spatial control that are difficult to achieve using conventional molding or casting approaches [22]. Extrusion-based printing has also been demonstrated for chitosan hydrogels, confirming the suitability of this biopolymer for the fabrication of three-dimensional hydrogel structures [24,25].

However, these approaches address the required functions separately rather than in combination. Printed thermochromic structures have generally been realized in thermoplastic or resin-based matrices [3,18], which are mechanically stiffer and are not water-rich, whereas thermo-responsive hydrogels with optical switching have typically been prepared as films or bulk specimens rather than as freestanding printed architectures [23]. Printed chitosan hydrogels, in turn, have been developed primarily as structural or scaffold materials without thermochromic functionality [24,25]. Consequently, previous work does not fully address the combined manufacturing requirements of an extrusion-printed, water-rich chitosan hydrogel that simultaneously incorporates passive thermochromic functionality and maintains sufficient dimensional stability for multilayer construction.

For extrusion-based hydrogel printing, material properties, extrusion conditions, and post-extrusion solidification cannot be considered independently. Ink flow behavior influences its passage through the nozzle, whereas nozzle dimensions, extrusion rate, and printing speed affect the amount and geometry of deposited material. After extrusion, sufficiently rapid stabilization is required to limit dimensional change and support subsequent layers [22,24,25,26,27]. Previous reviews of printed flexible sensors have emphasized that device performance depends not only on the sensing material itself but also on its flow behavior, on structural retention after deposition, and on the compatibility between the material system and the selected printing method [22,28]. This coupling is particularly important for chitosan-based thermochromic inks because delayed stabilization can result in filament expansion, loss of geometric fidelity, and collapse or distortion during multilayer deposition. These process-induced changes are not confined to geometry: strand diameter and interlayer contact govern the effective optical path and the area over which color develops, so variations in printing conditions propagate to the uniformity and legibility of the final thermochromic response. Therefore, an appropriate material–process strategy must combine controllable extrusion with rapid post-deposition stabilization while preserving the temperature-dependent optical functionality of the incorporated thermochromic pigments.

This study presents an integrated material–process strategy for additively manufactured thermochromic chitosan hydrogel indicators that provide passive, battery-free visual thermal readout. The specific objective of this study was to develop and evaluate a temperature-controlled coagulation-assisted extrusion-printing strategy that enables stable multilayer fabrication while preserving thermochromic functionality. We developed a syringe-pump-driven, extrusion-based direct ink writing platform coupled with a temperature-controlled coagulation bath, in which each deposited strand is stabilized by rapid in situ gelation at the moment of deposition rather than by post-deposition drying or additional crosslinking. This immediate stabilization limits filament expansion and preserves printed geometry, which in turn permits reproducible multilayer deposition and layer-resolved colorimetric readout of localized thermal states within a single construct. Because the readout is entirely optical, the resulting indicator reports temperature without electrical transduction, signal-conditioning circuitry, or a power source.

To achieve this objective, three aspects were systematically investigated. First, the effects of nozzle gauge, printing speed, and flow rate on printed strand dimensions and structural fidelity were evaluated. Second, the effects of coagulation bath temperature and chitosan concentration on the tensile behavior of the printed hydrogels were investigated. Third, the temperature-dependent colorimetric responses of two thermochromic pigment formulations were quantified in CIE L*a*b* color space and integrated into a multilayer architecture. Finally, the multilayer construct was used to demonstrate position-dependent colorimetric responses within a single 3D structure, supporting the potential of the developed platform for passive visual thermal indication.

## 2. Materials and Methods

### 2.1. Materials

Chitosan (from shrimp shells, practical grade, 417963-100G, degree of deacetylation: ≥75%, molecular weight: 190–375 kDa), lithium hydroxide (LiOH, 442410-100G-A), urea (U5128-1KG), and potassium hydroxide (KOH, 484016-1KG) were purchased from Sigma-Aldrich (St. Louis, MO, USA). Deionized (DI) water was prepared in the laboratory. The thermochromic microcapsule pigments used in the thermochromic hydrogel ink were obtained from Nano I&C (Asan, Republic of Korea). These microcapsules have a size distribution of 1–5 μm and operate based on a leuco dye system comprising an electron-donating leuco dye, an electron-accepting developer, and an internal organic solvent. Their color change mechanism relies on the temperature-dependent phase transition of the internal organic solvent, which induces a reversible acid-base equilibrium shift (electron donation/acceptance) in the dye to produce visual color shifts. These included B28241024 (blue, activated at 28 °C), Y33241024 (yellow, activated at 33 °C), and R38220713 (red, activated at 38 °C).

### 2.2. Preparation of Chitosan-Based Thermochromic Hydrogel Inks

Chitosan hydrogel ink. The preparation of chitosan hydrogel ink was adapted from previous reports [24,25]. Specifically, chitosan powder was dissolved in an alkaline solvent. The alkaline solvent comprised LiOH, urea, KOH, and DI water at a mass ratio of 5:8:7:80.5. Chitosan powder was added to the alkaline solvent at different concentrations (3.5, 4.0, and 4.5 wt%) and thoroughly stirred at room temperature (RT) until a homogeneous mixture was obtained. Unless otherwise specified, the 4.0 wt% formulation was used as the default composition throughout the study. The resulting chitosan hydrogel ink was stored at –19 °C to –15 °C for 6 h until fully frozen. Subsequently, the frozen hydrogel thawed at RT until it completely melted. The freeze–thaw cycle was repeated twice to ensure uniform dispersion. This process is essential to disrupt the existing hydrogen bond networks between chitosan chains that hinder dissolution at room temperature and to form new hydrogen-bonded complexes with the alkali solvent. This specific two-cycle protocol was empirically determined as optimal; a single cycle occasionally resulted in incomplete dissolution and microscopic aggregation, whereas additional cycles beyond two yielded no further improvements in ink homogeneity or printability, merely increasing the total processing time. All hydrogel inks were freshly prepared before their use in the experiments.

Thermochromic hydrogel ink. Thermochromic microcapsule pigments were added to the prepared chitosan hydrogel ink to fabricate temperature-responsive hydrogel ink. The thermochromic hydrogel ink was formulated by mixing chitosan hydrogel ink with blue, yellow, and red thermochromic pigments at mass ratios of 20:0.04:0.08:0.04 for the 1st formulation and 20:0.25:2.5:0.25 for the 2nd formulation. The three pigments were selected based on their nominal activation temperatures of 28 °C (blue), 33 °C (yellow), and 38 °C (red), thereby providing overlapping thermochromic transitions across the intermediate temperature range of interest. The 1st formulation was used as the initial composition, whereas the pigment fractions in the 2nd formulation were adjusted, particularly by increasing the fraction of the 33 °C-activated yellow pigment, to increase visually distinguishable color variation within the intermediate temperature range. By adjusting the ratio of the thermochromic pigments, the temperature-dependent color-transition profile of the thermochromic hydrogel indicator was modulated. The mixture was stirred thoroughly at RT until homogeneous, followed by vacuum treatment, during which air bubbles were removed. For consistent ink preparation, the same mixing, homogenization, and degassing sequence was applied to each batch, and freshly prepared inks were used for subsequent printing experiments.

### 2.3. Development of a Syringe-Pump-Driven Hydrogel Printing Platform with a Multi-Nozzle Setup

For the experiments, a commercially available filament-based 3D printer (Ender-3 Neo, Creality, Shenzhen, China) was modified to enable hydrogel printing. The original extruder system was replaced with a syringe pump (NE-300 Just Infusion™, New Era Pump Systems Inc., Farmingdale, NY, USA), which was designed to allow precise control of the discharge rate, enabling the use of thermochromic hydrogel ink instead of filament materials.

A completely sealed flow path and nozzle system was constructed using a 10 mL luer-lock plastic syringe, tubing, syringe luer-lock connectors, luer-lock tubing connectors, and industrial luer-lock plastic needles. Needles of various gauges, including 19G, 21G, and 23G, with inner diameters of 0.65, 0.51, and 0.33 mm, respectively, were used. This setup was configured to provide stable and uniform control of discharge flow without any viscous fluid leakage or pressure loss during extrusion. The original printhead and cover components were removed to mount the newly configured nozzle system onto the printhead of the 3D printer. Then, a new printhead was designed, fabricated using a fused deposition modeling 3D printer (P1S, Bambu Lab, Shenzhen, China), and secured with screws. To enable easy detachment and stable fixation, particularly for the replacement of damaged parts, the use of hydrogel-based inks with different viscosities, changes in needle size, and cleaning of clogged needles, the nozzle was fastened to the printhead using bolts and nuts. A multi-nozzle setup was also configured using the above approach to enable multi-material printing. This setup comprised two nozzle assemblies and two syringe pumps.

To precisely stack the hydrogel according to the design, proper in situ gelation must occur. Immediate in situ gelation upon hydrogel extrusion is essential to prevent the overall shape from collapsing and to support subsequent layers robustly, thereby forming a stable printed structure [25]. Therefore, in this study, an underwater printing system was configured, where the hydrogel was directly extruded into a heated coagulation bath to induce immediate in situ gelation, thereby supporting structure formation. The coagulation bath consisted solely of DI water without the addition of any coagulant or other chemical component. During printing, the nozzle temperature was maintained at RT, whereas the coagulation bath temperature was maintained within the range of 50–70 °C. Unless otherwise specified, a coagulation bath temperature of 60 °C was used as the default condition for all experiments, except for temperature-dependent evaluations. After printing, the samples were immersed in a coagulation bath at the printing temperature for 30 min to achieve complete gelation. Subsequently, the printed samples were washed ten times with fresh DI water to remove residual alkali. To evaluate the removal of residual alkali during the washing procedure, the pH of the immersion water was measured at three stages. Each specimen (4.0 wt% chitosan) was immersed in 10 mL of DI water in a sealed vial, and the pH was recorded immediately after printing, after washing, and after five days of immersion in the same DI water to assess cumulative alkali release. The pH was measured using a digital pH meter (PH-8000, ETI, Worthing, United Kingdom) on five specimens per stage. Values are reported as mean ± standard deviation. The DI water used throughout had a pH of 7.2. Finally, for further chitosan hydrogel characterization, the structures were stored in DI water at 8 °C.

The water content and swelling ratio of the printed hydrogels were determined gravimetrically. Washed specimens (12 mm × 12 mm × 2 mm) were weighed in the as-prepared wet state (W_wet_), dried in an oven at 60 °C for 12 h and weighed again (W_dry_), and then immersed in DI water for 24 h and weighed after removing surface water with a lint-free wipe (W_rehydration_). The water content and swelling ratio were calculated as (W_wet_ − W_dry_)/W_wet_ × 100 (%) and (W_rehydration_ − W_dry_)/W_dry_, respectively. Five specimens were measured per condition, and the results are reported as mean ± standard deviation. Cross-sectional morphologies were observed by scanning electron microscopy (SNE-Alpha, SEC co., Ltd., Suwon, Republic of Korea). Specimens dried under the same conditions were fractured, mounted, and sputter-coated before imaging.

### 2.4. Control of Die Swelling

To investigate and control die swelling during extrusion, samples were printed into a heated coagulation bath using three nozzle gauges: 19G, 21G, and 23G. The printing speeds were varied between 5 and 35 mm/s, and the flow rates were adjusted from 0.15 to 0.25 mL/min to assess their effects on the hydrogel ink. The extent of die swelling was evaluated using an optical microscope (LV150N, Nikon, Tokyo, Japan).

### 2.5. Tensile Testing

To evaluate the mechanical behavior of the printed chitosan hydrogel structures, uniaxial tensile tests were conducted on rectangular prism-shaped specimens using a universal testing machine at a crosshead speed of 40 mm/min. The printed specimens had dimensions of approximately 30 mm × 3.95 mm × 2.4 mm, with a designated gauge length (working part) of 2.02 mm between the grips; the short gauge length was necessary because the highly hydrated specimens were prone to slipping at longer working lengths. To ensure the reproducibility of the mechanical characteristics, 5 independent replicate samples (*n* = 5) were tested for each experimental condition.

All tensile tests were conducted immediately after gelation without additional storage. To examine the effect of gelation temperature on tensile properties, experiments were conducted at 50 °C, 60 °C, and 70 °C. Chitosan hydrogel inks with chitosan concentrations of 3.5, 4.0, and 4.5 wt% were used to investigate the influence of chitosan content on the mechanical properties of the printed hydrogel constructs.

A universal testing machine (QM100S, QMESYS, Uiwang, Republic of Korea) was used at a constant tensile speed of 40 mm/min. During testing, the force and displacement data were recorded and converted to stress–strain curves. Stress and strain were calculated using the initial cross-sectional area and gauge length of each specimen, respectively. The ultimate tensile strength (UTS) was defined as the maximum stress before fracture.

Environmental mechanical stability was additionally evaluated using molded specimens (30 mm × 3.95 mm × 2.4 mm, *n* = 5). The specimens were subjected to five heating–cooling cycles between 60 °C for 5 min and room temperature for 5 min under hydrated conditions. A separate group underwent partial dehydration at 60 °C for 1 h followed by rehydration in 30 °C water for 1 h. Tensile testing was subsequently performed under the same conditions described above.

### 2.6. Analysis of Thermochromic Behavior

The temperature-dependent colorimetric behavior of the thermochromic hydrogel ink was evaluated using a lattice-patterned thermochromic hydrogel indicator printed via an extrusion-based 3D printing platform. Two formulations were prepared by incorporating thermochromic microcapsule pigments into 20 g of chitosan-based hydrogel ink. The 1st formulation contained 0.04 g of blue, 0.08 g of yellow, and 0.04 g of red pigments. The 2nd formulation contained 0.25 g of blue, 2.5 g of yellow, and 0.25 g of red pigments to enhance the color contrast in the mid-temperature range.

The printed thermochromic hydrogel indicators were placed on a temperature-controlled hot plate (MSH-20D, DAIHAN Scientific, Wonju, Republic of Korea) and exposed to temperatures ranging from 26 to 54 °C in 1–2 °C increments. At each setpoint, the samples were equilibrated to allow complete pigment activation or deactivation. Photographs documenting the sequential color transitions were acquired using a smartphone camera (Galaxy A34 5G, Samsung, Suwon, Republic of Korea) under the ambient fluorescent ceiling illumination of the laboratory, without supplementary light. Images were captured in automatic mode at a fixed lens-to-sample distance of 10 cm and at normal incidence, with the samples placed on a silicon wafer substrate. No color calibration or manual white-balance correction was applied. These photographs are provided for qualitative documentation of the color transitions.

For quantitative analysis, colorimetric measurements were performed using a spectrophotometer (CM-26d, KONICA MINOLTA, Tokyo, Japan). At five representative temperatures (26, 31, 36.5, 38, and 54 °C), the CIE L*a*b* parameters (L*, a*, b*) were recorded in SCI mode under D65 illumination. The measured CIE L*a*b* data were used for direct comparison of L* values and a*–b* chromaticity maps between the two formulations. Because the spectrophotometer employs an internal D65 light source and an integrating sphere geometry, the CIE L*a*b* values are independent of ambient lighting conditions and viewing angle. All quantitative colorimetric comparisons in this study are therefore based on these measurements rather than on photographs.

Each specimen was measured at all target temperatures, so that the temperature-dependent color change was tracked on the same specimen (*n* = 5 specimens per formulation). The color difference between adjacent temperature states was evaluated using the CIE 1976 color-difference formula (CIE76) [29]:

(1) $$\Delta E_{ab}^{*}=\sqrt{{\left(\Delta L^{*}\right)}^{2}+{\left(\Delta a^{*}\right)}^{2}+{\left(\Delta b^{*}\right)}^{2}}$$

where $\Delta L^{*}$, $\Delta a^{*}$, and $\Delta b^{*}$ denote the differences in the respective coordinates between two temperature states. $\Delta E_{ab}^{*}$ was calculated for each specimen individually and is reported as mean ± standard deviation.

Cycling stability was evaluated by repeatedly heating and cooling the indicators between 26 and 54 °C for 50 cycles. CIE L*a*b* coordinates were measured before cycling and after 50 cycles at 26, 31, 36.5, 38, and 54 °C during both heating and cooling, and the color difference between the heating and cooling paths at each temperature was taken as the thermal hysteresis, calculated using Equation (1). The specimen mass was recorded before and after cycling. Response and recovery behavior was analyzed after placing an indicator on a hot plate preheated to 54 °C and after returning it to room temperature (*n* = 5).

### 2.7. Fabrication and Temperature-Controlled Evaluation of Dual-Ink Thermochromic Hydrogel Indicator

A dual-ink thermochromic hydrogel indicator with a lattice structure was fabricated in a dual-nozzle configuration using the syringe-based multi-nozzle printing platform. A multilayer lattice design was adopted, in which the bottom lattice frame (approximately 20 mm × 20 mm × 1.2 mm) was printed first, followed by the central block-like core (11 mm × 11 mm × 3.6 mm) and the top lattice layers (11 mm × 11 mm × 1.2 mm). The bottom lattice frame and top lattice layers were printed using a thermochromic hydrogel ink, whereas the block-like core region was printed using a non-thermochromic chitosan-based hydrogel ink. The thermochromic hydrogel ink followed the 1st formulation, whereas the chitosan hydrogel ink without thermochromic pigments served as the non-thermochromic ink.

For temperature-controlled tests, the indicators were placed on a temperature-controlled hot plate and the plate temperature increased stepwise from 26 to 54 °C. At each step, the ambient air and lattice surface temperatures were monitored and recorded using a digital thermometer. Images were acquired at plate temperatures of 26, 30, and 54 °C, which were selected as the states showing the most pronounced color contrast for the 1st formulation. After 30 s at each target temperature, images of the indicators were acquired using the same camera, illumination, and working distance described in Section 2.6.

The temperature of each thermochromic layer was measured using a digital thermistor probe thermometer (LUAZ-PT, Luaz, Chengdu, China; resolution 0.1 °C, accuracy ±1 °C, stainless-steel 304 probe). The probe tip was placed in contact with the surface of the upper or lower lattice layer, and the reading was recorded 30 s after placement. The ambient air temperature above the construct was measured with the same instrument. Measurements were performed on five independently printed constructs for each plate temperature, and the results are reported as mean ± standard deviation.

### 2.8. Statistical Analysis

Image processing and quantitative measurements were performed using ImageJ (version 1.53e; National Institutes of Health, Bethesda, MD, USA). Data processing and statistical analyses were performed using Microsoft Excel (Microsoft 365, version 2608; Microsoft Corporation, Redmond, WA, USA). All data are presented as means with standard deviations (mean ± SD). For comparisons involving more than two groups, such as variations in coagulation bath temperatures and chitosan concentrations, statistical significance was determined using a one-way analysis of variance (ANOVA) followed by Tukey’s post hoc test. For comparisons between two independent groups, a two-tailed Student’s *t*-test was applied. Statistical significance was defined as * *p* < 0.05, ** *p* < 0.01, and *** *p* < 0.001. Each experimental condition was tested with five independent replicates (*n* = 5), unless otherwise stated.

## 3. Results and Discussion

### 3.1. Additive Manufacturing Platform and System Configuration for Thermochromic Hydrogel Indicator Fabrication

Figure 1 shows the overall configuration of the additive manufacturing platform used to fabricate thermochromic hydrogel indicators. The thermochromic hydrogel ink was prepared by uniformly dispersing blue, yellow, and red thermochromic microcapsule pigments in a chitosan-based hydrogel precursor, enabling reversible, multistage color transitions in response to temperature changes (Figure 1A(i)). During printing, the precursor was extruded into a heated coagulation bath placed on the printing bed, where a temperature-driven phase transition induced rapid gelation and helped maintain filament integrity and shape fidelity (Figure 1A(ii)). The printed multilayer constructs exhibited reversible color transitions over repeated thermal cycles, supporting their use as visually readable thermochromic hydrogel indicators.

The customized hardware integrates a dual-syringe pump system with an x–y–z motion stage positioned above the coagulation bath (Figure 1B). This configuration enables coordinated control of extrusion flow and nozzle motion, facilitating the stable deposition of inks with different viscosities and mitigating nozzle clogging and interlayer distortion. A commercial desktop 3D printer was mechanically modified to incorporate a temperature-controlled coagulation bath, a heated printing bed, and dual-syringe pump modules, providing a versatile platform for hydrogel processing. The modular design further enables the simultaneous or sequential deposition of multiple thermochromic hydrogel inks, allowing the fabrication of customizable, multicolor sensing architectures.

The printhead can operate in single- or multi-nozzle modes for spatially resolved deposition of distinct hydrogel inks within a single printing sequence (Figure 1C). Each nozzle was independently connected to its corresponding syringe pump, allowing rapid material switching without manual replacement. A built-in Z-axis sensor continuously monitors and adjusts the printing height to maintain consistent layer thickness and reduce collapse-related defects, thereby improving interlayer alignment and print-to-print reproducibility. Overall, the integrated system enables reproducible fabrication of multilayer thermochromic hydrogel indicators with localized color-responsive features, providing a fabrication basis for wearable and, prospectively, skin mountable thermal indicators.

### 3.2. Mitigation of Die Swelling and Printing Fidelity in a Thermochromic Chitosan Hydrogel Printing System

The die swelling behavior of the thermochromic chitosan hydrogel during extrusion was quantitatively analyzed to identify key process parameters for dimensionally stable printing (Figure 2). The ink underwent shear deformation within the nozzle and then viscoelastic recovery at the outlet, producing an extrudate diameter (D_out_) greater than the inner diameter (D_in_) of the nozzle, a hallmark of viscoelastic die swelling (Figure 2A). The magnitude of swelling depends on the flow velocity (v) and the viscoelastic relaxation time of the ink. To mitigate this effect, a heated coagulation bath was used to trigger rapid thermal gelation immediately upon exit, thereby restricting radial expansion and promoting early stabilization of the strand diameter. Optical imaging confirmed that the printed strands followed the designed toolpaths with uniform spacing, producing stable patterns without obvious collapse or fusion (Figure 2B). High-magnification optical micrographs further revealed single strands with a nearly uniform diameter (~700 µm) and smooth surfaces, with no apparent surface defects.

To quantitatively support these observations and define a process window for strand diameter control, the nozzle gauge (19G, 21G, and 23G), printing speed (5–35 mm/s), and flow rate (0.15–0.25 mL/min) were systematically varied. The coagulation bath temperature was fixed at 60 °C for all conditions. As the nozzle inner diameter decreased, the strand diameter also decreased, and within each nozzle gauge, increasing the printing speed led to a further reduction in diameter (Figure 2C). To evaluate this behavior, the die-swelling ratio (defined as extrudate diameter divided by nozzle inner diameter) was calculated (Figure S1). At a printing speed of 5 mm/s, the measured strand-to-nozzle diameter ratios were 2.13, 2.72, and 3.70 for the 19G, 21G, and 23G nozzles, respectively. As the printing speed increased, the ratio progressively decreased, reaching approximately 1.0 at intermediate speeds (10–15 mm/s for 19G and 21G; 20 mm/s for 23G), beyond which mechanical drawing (ratio < 1.0) occurred. At a fixed volumetric flow rate, increasing the printing speed reduces the volume deposited per unit path length, contributing to the decrease in strand diameter. Rapid gelation in the heated coagulation bath may additionally help stabilize the deposited geometry. In contrast, increasing the flow rate resulted in a proportional increase in strand diameter due to the larger extrusion volume per unit time, whereas lower flow rates yielded smaller strand diameters (Figure 2D). These results indicate that the interplay among printing speed, flow rate, and nozzle gauge governs the degree of die swelling, enabling predictable, parameter-dependent control of the strand diameter.

To further assess 3D printing fidelity, benchmark geometries were fabricated under optimized conditions. In each panel, the computer-aided design (CAD) model (left) and the printed construct (right) closely matched. The stepped pyramid exhibited collapse-free vertical stacking with a progressive, uniform reduction in layer width (Figure 2E), whereas the lattice maintained consistent cross-bridging and an open-pore architecture across all layers, faithfully reproducing the designed pattern (Figure 2F). Dimensional deviations were quantified by comparing the base widths of the printed constructs with their CAD dimensions. The printed stepped pyramid had a base width of approximately 13 mm, compared with the CAD value of 14.4 mm, corresponding to an absolute relative dimensional error of 9.7%. The printed lattice had a base width of approximately 10.5 mm, compared with the CAD value of 11.255 mm, corresponding to an error of 6.7%. These errors were calculated as the absolute difference between the printed and CAD widths divided by the CAD width. Collectively, these results indicate that the process extends beyond planar patterns to free-form 3D architectures, supporting geometric reproducibility and shape retention under the optimized conditions.

Beyond dimensional control, these process parameters also determine the geometric basis of the subsequent colorimetric response. In the present indicators, color is developed by thermochromic microcapsules dispersed throughout the printed strand, so the strand diameter and the strand-to-strand spacing set the projected area over which color is developed within the lattice geometry. At fixed spacing, a larger strand diameter increases the colored fraction of the projected area and correspondingly reduces the open-pore fraction. Because die swelling was suppressed by in situ gelation in the 60 °C coagulation bath, the strand diameter obtained under a given parameter set remained consistent across the printed area, providing a spatially uniform geometric basis for the colorimetric measurements described in Section 3.4.

These findings demonstrate predictable, parameter-dependent control of die swelling and the strand diameter through systematic tuning of extrusion parameters. The heated coagulation bath provided a consistent thermal gelation environment that preserved structural stability. Overall, these results show that fine-tuning process parameters within a simple syringe-based extrusion setup minimizes strand diameter variation and structural deformation, enabling reproducible and dimensionally stable printing.

### 3.3. Tensile Properties of Chitosan Hydrogels as a Function of Coagulation Bath Temperature and Chitosan Concentration

After thermal gelation, the printed chitosan hydrogel specimens retained the intended rectangular geometry, with close agreement between the design model (left) and the fabricated construct (right) (Figure 3A). The tensile testing configuration is shown in Figure 3B, illustrating the initial state (ε = 0) and the maximum extension at fracture (ε = ε_max_).

The coagulation bath temperature affected the tensile behavior of the hydrogels (Figure 3C). UTS increased from 0.155 ± 0.006 MPa at 50 °C to 0.172 ± 0.009 MPa at 60 °C, a significant increase relative to the 50 °C condition, whereas the 70 °C condition showed no additional gain over 60 °C (0.170 ± 0.013 MPa) (Figure 3D). Consistent trends were observed in the fracture strain and toughness derived from the averaged stress–strain curves. The fracture strain increased from 184% at 50 °C to 230% at 60 °C, followed by a slight decrease to 217% at 70 °C. Similarly, the toughness reached a maximum at 60 °C (0.269 MJ/m^3^), compared with 0.215 MJ/m^3^ at 50 °C and 0.247 MJ/m^3^ at 70 °C.

To examine whether these differences in tensile behavior were accompanied by differences in the resulting network, the water content and swelling ratio of the printed hydrogels were measured (Figure S2A). Both quantities were highest at 60 °C, with values of 94.21 ± 0.11% and 15.90 ± 0.29 and were significantly lower at both 50 °C (93.42 ± 0.24%, 13.52 ± 0.66; *p* < 0.001 for both) and 70 °C (93.67 ± 0.29%, 14.57 ± 0.80; *p* < 0.01 and *p* < 0.05, respectively). The higher values at 60 °C indicate a network that retains more water, consistent with the improved tensile performance at this condition. Although the UTS values at 60 and 70 °C were not statistically different, both the water content and the swelling ratio differed significantly between them, indicating that the coagulation bath temperature affects the network even where this difference is not resolved in the tensile response [30]. These trends are consistent with a temperature-dependent balance in the coagulation process. At 50 °C, slow coagulation leads to a more loosely stabilized structure. At 60 °C, the elevated bath temperature may enhance molecular mobility during the early stage of gelation, allowing the network to develop more uniformly [31]. At 70 °C, gelation may proceed too rapidly for uniform network development, which has been reported to compromise the mechanical performance of hydrogels [32]. Collectively, these results indicate that coagulation bath temperature is a key process parameter governing the balance between gelation rate and network uniformity, with 60 °C providing the best overall tensile performance among the tested conditions.

Chitosan concentration produced pronounced differences in tensile behavior (Figure 3E). The 3.5 wt% chitosan specimens exhibited the lowest UTS (0.081 ± 0.009 MPa), whereas the 4.0 wt% and 4.5 wt% formulations were significantly higher (0.172 ± 0.009 MPa and 0.165 ± 0.011 MPa, respectively) and did not differ significantly from each other (Figure 3F). The fracture strain and toughness, however, distinguished these two formulations: both were highest at 4.0 wt% (230% and 0.269 MJ/m^3^), and lower at 4.5 wt% (162%, 0.191 MJ/m^3^) and 3.5 wt% (129%, 0.082 MJ/m^3^). As reflected by the initial slope of the stress–strain curves, the 4.5 wt% specimens were stiffer than the 4.0 wt% specimens but fractured at a lower strain, indicating a more brittle response. Thus, the 4.0 wt% formulation combined a high tensile strength with greater extensibility and energy absorption, providing the most balanced tensile performance among the tested concentrations.

The water content and swelling ratio followed the same trend (Figure S2B). The 4.0 wt% specimens exhibited the highest values (94.22 ± 0.12% and 15.51 ± 0.40), whereas the 4.5 wt% specimens were significantly lower (93.17 ± 0.23%, 12.90 ± 0.46; *p* < 0.001 for both); the 3.5 wt% specimens were comparable to the 4.0 wt% specimens (94.06 ± 0.08%, 14.89 ± 0.55). Cross-sectional SEM images of the dried hydrogels were consistent with these measurements (Figure S3): the 3.5 and 4.0 wt% specimens showed open porous structures, whereas the 4.5 wt% specimens appeared densely packed with few resolvable pores. The reduced water content and swelling capacity at 4.5 wt% therefore reflect a denser network at higher polymer content, which is consistent with the increased stiffness and reduced extensibility observed in the tensile tests. In summary, increasing chitosan concentration did not produce a monotonic improvement in tensile performance; instead, a trade-off between stiffness and extensibility emerged with increasing polymer content.

Taken together, these results demonstrate that coagulation bath temperature and chitosan concentration are effective parameters for tuning the mechanical behavior of chitosan hydrogels. Such mechanically tunable behavior can be advantageous for conformal contact with curved or deformable surfaces such as skin.

In addition, to evaluate the removal of residual alkali during the washing procedure, the pH of the immersion water was measured at three stages (Figure S4). The as-printed samples exhibited an alkaline pH of approximately 10.8. After ten washing cycles with fresh DI water, the pH decreased substantially to 7.8, indicating effective removal of most soluble alkaline components. After five days of immersion without replacement of the water, the pH increased moderately to about 8.8, suggesting gradual release of a limited amount of residual alkaline species from the hydrogel. These results indicate that repeated DI-water washing markedly reduces the alkalinity of the printed hydrogel; however, the observed increase during prolonged immersion also highlights the need for further optimization of washing or encapsulation strategies for applications involving direct and prolonged skin contact.

Pigment leaching was also assessed by measuring the color of the immersion water of specimens stored for one year (Table S1). The color difference between the immersion water and fresh DI water was $\Delta E_{ab}^{*}$ = 0.31, below the threshold at which a color difference becomes perceptible to an observer. Release of the thermochromic pigment into the surrounding water was therefore below the detection limit of the measurement, indicating that the pigments remained retained within the hydrogel network during prolonged immersion.

To further evaluate the practical durability of the thermochromic hydrogels, mechanical stability was assessed under cyclic environmental conditions (Figure S5). For these tests, standardized hydrogel specimens (30 mm × 3.95 mm × 2.4 mm) were prepared using a custom mold to ensure consistent baseline dimensions. Although the absolute tensile values of these bulk-cast specimens differ from the layer-by-layer 3D-printed constructs evaluated earlier, they provide a controlled baseline for assessing the relative environmental stability of the hydrogel network itself.

When subjected to 5 continuous heating (60 °C) and cooling (room temperature) cycles in a hydrated environment, the hydrogels retained an ultimate tensile strength (UTS) of 0.258 ± 0.019 MPa, approximately 92.4% of the initial value (0.279 ± 0.023 MPa). This indicates that the repeated thermal transitions did not induce substantial fatigue or structural degradation within the polymer network.

Stability against moisture fluctuation was also tested via a sequential partial dehydration (60 °C, 1 h) and accelerated rehydration (30 °C, 1 h) cycle. Following this cycle, the UTS increased to 0.559 ± 0.042 MPa. This increase is attributed to volumetric shrinkage and densification of the polymer network upon dehydration, which did not fully reverse within the rehydration period applied. Although no structural failure occurred, this indicates that applications requiring precise long-term dimensional retention would need to account for such moisture-induced changes. 

### 3.4. Temperature-Dependent Colorimetric Response of Thermochromic Indicators

The thermochromic hydrogel indicator exhibited temperature-dependent color transitions over 26–54 °C, with visually distinguishable changes across the tested range (Figure 4A). At 26 °C, the entire structure appeared violet. As the temperature increased to 28–30 °C, the color gradually shifted to orange tones. At 31 °C, the indicator exhibited an orange appearance. At 36.5 °C, the color transitioned to an orange-pink tone, and at 38 °C it shifted further toward pink. Above 42 °C, the indicator progressively lost chroma, and by 54 °C, it appeared nearly white.

Quantitative colorimetric analysis supported these visual observations (Figure 4B). In the a*–b* chromaticity map, the 26 °C sample was located in the positive a* and negative b* region, corresponding to a violet appearance along the red−blue direction. From 31 to 38 °C, a* values increased, whereas b* values gradually decreased. This shift indicates a subtle hue transition from orange to orange-pink and then to pink. In contrast, the 54 °C sample was positioned close to the origin with low a* and b* values, corresponding to a low-chroma, near-white appearance. L* increased from 26 to 31 °C, varied slightly across the 31–38 °C range, and reached its highest value at 54 °C. Together, these results show that the 1st formulation produced a consistent temperature-dependent chromatic trajectory across the tested range, while the 31–38 °C samples occupied a relatively narrow region in CIE L*a*b* color space.

To examine the effect of changes in the thermochromic pigment formulation on the temperature-dependent chromatic response, a 2nd formulation was tested. Particular attention was given to how adjacent color states were distributed in CIE L*a*b* color space within the skin-relevant intermediate temperature range. The thermochromic hydrogel indicator fabricated using the 2nd formulation exhibited distinct temperature-responsive color transitions over 26–54 °C (Figure 5A). At 26 °C, the indicator exhibited an olive-toned appearance. As the temperature increased to 28–35 °C, the color shifted to a warm yellow-orange tone. At 36.5 °C, the indicator exhibited a yellowish pink appearance, which shifted to a more saturated pink at 38 °C. At higher temperatures (≥48 °C), the color progressively lost chroma, and by 54 °C, the indicator appeared nearly white.

Quantitative colorimetric analysis was consistent with the visual color transitions observed in Figure 5A (Figure 5B). In the a*–b* chromaticity map, the 26 °C sample exhibited a low positive a* value and a positive b* value, placing it in a yellow-containing region; visually, it appeared close to an olive tone. The samples at 31, 36.5, and 38 °C were distributed in the positive a* region and exhibited a broader distribution in CIE L*a*b* color space.

Specifically, the 31 °C sample was located in a higher-b* region, whereas the 36.5 and 38 °C samples shifted toward lower b* values while maintaining positive a* values, consistent with the visual shift from yellow-orange at 31 °C to yellowish pink at 36.5 °C and to a more saturated pink at 38 °C. The 54 °C sample was positioned near the origin with low a* and b* values, corresponding to its low-chroma, near-white appearance. In parallel, L* increased across the sampled temperatures from 26 to 54 °C, indicating progressive brightening with increasing temperature. These results show that the 2nd formulation produced a broader distribution of the 31, 36.5, and 38 °C states in CIE L*a*b* color space.

To quantify the distinguishability between adjacent temperature states, the color difference ($\Delta E_{ab}^{*}$) between neighboring measured states was calculated (Table S2). For the 1st formulation, the values within the 31–38 °C range were small, at 2.59 ± 0.47 between 31 and 36.5 °C and 1.57 ± 1.26 between 36.5 and 38 °C. According to the perceptibility classification reported for CIELAB color differences [33], values in this range correspond to differences that are noticeable only to an experienced observer or to a moderately experienced observer, rather than differences that are unambiguously visible. For the 2nd formulation, the corresponding values were 10.19 ± 0.28 and 7.80 ± 0.34, both exceeding the threshold above which two colors are perceived as clearly distinct. Adjusting the pigment formulation therefore markedly improved the colorimetric distinguishability of neighboring thermal states within this range, consistent with the broader distribution of color states described above.

Taken together, these results demonstrate that changes in thermochromic pigment formulation provide a practical means of modulating the temperature-dependent chromatic response of the hydrogel indicator. This tunability supports a simple, battery-free, color-change-based hydrogel indicator for skin-relevant temperature detection and stepwise thermal-alarm applications [3,14,34].

The reversibility and repeatability of the colorimetric response were evaluated for the 1st formulation over 50 heating–cooling cycles between 26 and 54 °C (*n* = 5). After 50 cycles, the CIE L*a*b* coordinates measured during heating were essentially unchanged from their initial values, with color differences of $\Delta E_{ab}^{*}$ = 0.15–0.91 across the five temperature states (Figure S6, Table S3). The color differences between the heating and cooling paths at the 50th cycle were similarly small ($\Delta E_{ab}^{*}$ = 0.28–0.83), indicating negligible thermal hysteresis. All of these values lie below the threshold at which a color difference becomes perceptible, and are smaller than the specimen-to-specimen standard deviations of the individual L*, a*, and b* coordinates, indicating that both the cycling-induced change and the hysteresis were within the measurement variability.

The distinguishability between adjacent temperature states was also preserved. After 50 cycles, the $\Delta E_{ab}^{*}$ values between neighboring states (9.11 ± 1.17, 1.88 ± 0.68, 1.31 ± 1.47, and 9.25 ± 0.99 for the 26 to 31, 31 to 36.5, 36.5 to 38, and 38 to 54 °C transitions) were comparable to those measured before cycling (Table S4), confirming that repeated thermal cycling did not degrade the temperature-dependent color response. The specimen mass was 4.75 ± 0.31 g before cycling and 4.59 ± 0.12 g after 50 cycles, a difference that was not statistically significant (*p* = 0.33), indicating that the water content was retained throughout the test.

The response behavior was evaluated by placing an indicator on a hot plate preheated to 54 °C (Figure S7). The color change proceeded in two stages, likely reflecting the sequential thermochromic transitions associated with the different nominal activation temperatures of the three pigments. The initial violet-to-pink transition, associated with the 28 °C-activated blue pigment, was completed within approximately 2 s, whereas the subsequent change toward the near-white state, involving the 33 and 38 °C-activated pigments, was complete within approximately 15 s. Upon returning the indicator to room temperature, the original color was recovered within approximately 8.5 s.

### 3.5. Layer-Resolved Colorimetric Readout in a Multilayer Thermochromic Hydrogel Indicator

To demonstrate spatially resolved thermal indication within a multilayer indicator, a stacked lattice construct was fabricated using the 1st formulation. The upper and lower lattice layers were printed with thermochromic hydrogel ink, while an intermediate chitosan hydrogel layer was introduced to spatially separate the two thermochromic layers (Figure 6A). In this architecture, the lower thermochromic lattice layer was positioned closer to the heat source, whereas the upper layer was more strongly influenced by the surrounding air, placing the two layers in different thermal environments. The intermediate chitosan hydrogel layer served as a mechanical support and spacer and did not directly contribute to temperature-dependent color change. Because each deposited strand gelled immediately upon entering the coagulation bath, the lower lattice layer retained its open-pore geometry while supporting the intermediate spacer and the upper lattice layer without visible collapse, and the stacked construct could be handled as a single self-supporting object. This shape retention preserved the vertical separation between the two thermochromic layers, without which the position-dependent color states observed in Figure 6B could not have been resolved.

When exposed to different ambient and plate temperature conditions (Figure 6B), the multilayer construct exhibited distinct color differences between the upper and lower thermochromic layers. Under ambient 23.2 °C and plate 26 °C conditions, both layers exhibited violet-toned appearances, indicating that the construct remained in a low-temperature state. Under ambient 26.5 °C and plate 30 °C conditions, the lower lattice layer in direct contact with the plate shifted to a warm orange tone, whereas the upper layer retained a more violet-like appearance. This contrast indicates that a layer-to-layer height difference on the order of a few millimeters can produce distinguishable colorimetric responses between the upper and lower layers. Under ambient 42.7 °C and plate 54 °C conditions, the lower lattice layer became nearly white, whereas the upper layer retained a pink-toned appearance. These observations demonstrate that the multilayer design enables layer-resolved colorimetric readout of localized thermal states within a single 3D structure.

Direct temperature measurements confirmed that the observed color differences corresponded to an actual thermal gradient within the construct (Table S5). The lower layer was consistently warmer than the upper layer, and the difference increased with plate temperature, from 1.6 °C at a plate temperature of 26 °C (25.2 ± 0.8 °C versus 23.6 ± 0.8 °C) to 2.7 °C at 30 °C (29.1 ± 1.0 °C versus 26.4 ± 0.9 °C) and 8.9 °C at 54 °C (52.6 ± 1.6 °C versus 43.7 ± 0.7 °C).

The measured temperatures were consistent with the chromatic states of the 1st formulation shown in Figure 4. At a plate temperature of 26 °C, both layers remained below 26 °C and retained the violet appearance of that range. At 30 °C, the lower layer reached 29.1 °C, matching the orange-toned state observed between 28 and 30 °C, whereas the upper layer at 26.4 °C remained violet. At 54 °C, the lower layer reached 52.6 °C, within the near-white range above 50 °C, and the upper layer at 43.7 °C corresponded to the pink state near 44 °C. 

This position-dependent color change was broadly consistent with the temperature-dependent chromatic behavior of the 1st formulation. Taken together, these results show that the multilayer indicator can distinguish localized temperature differences arising from positional variation within a 3D hydrogel structure through layer-resolved colorimetric readout. This finding supports the potential of this passive hydrogel indicator for battery-free, spatially resolved thermal indication.

## 4. Conclusions

This study demonstrates that immediate in situ gelation, enabled by a temperature-controlled coagulation bath, provides an effective manufacturing strategy for preserving dimensional fidelity during thermochromic chitosan hydrogel printing. Rapid gelation suppressed die swelling, with the die-swelling ratio, which ranged from 2.1 to 3.7 at 5 mm/s, decreasing to approximately 1.0 at intermediate printing speeds, enabling parameter-dependent control of strand diameter and reproducible multilayer fabrication without additional post-deposition stabilization steps. Coagulation bath temperature and chitosan concentration tuned the mechanical response of the printed hydrogels, with gelation at 60 °C yielding the highest ultimate tensile strength (0.172 ± 0.009 MPa) and a 4.0 wt% formulation providing the most balanced combination of strength and extensibility. Thermochromic pigment composition modulated chromatic separation across temperature states in CIE L*a*b* color space over 26–54 °C, and adjusting the formulation broadened the separation of the 31, 36.5, and 38 °C states, a range spanning normal skin temperature and the febrile range. Moreover, the multilayer construct demonstrated layer-resolved colorimetric readout within a single 3D structure, which distinguishes the present platform from planar thermochromic films and enables battery-free, spatially resolved thermal indication without electrical transduction or a power source. Future work will focus on quantitative rheological characterization of the printing inks to describe the extrusion and gelation behavior more rigorously and to support predictive selection of printing parameters. Long-term operational and repeated-use stability, readout reliability under diverse environmental conditions, and performance under practical skin-interfaced conditions also warrant systematic evaluation [3,12,14,20,34,35,36,37].

## Figures and Tables

**Figure 1:**
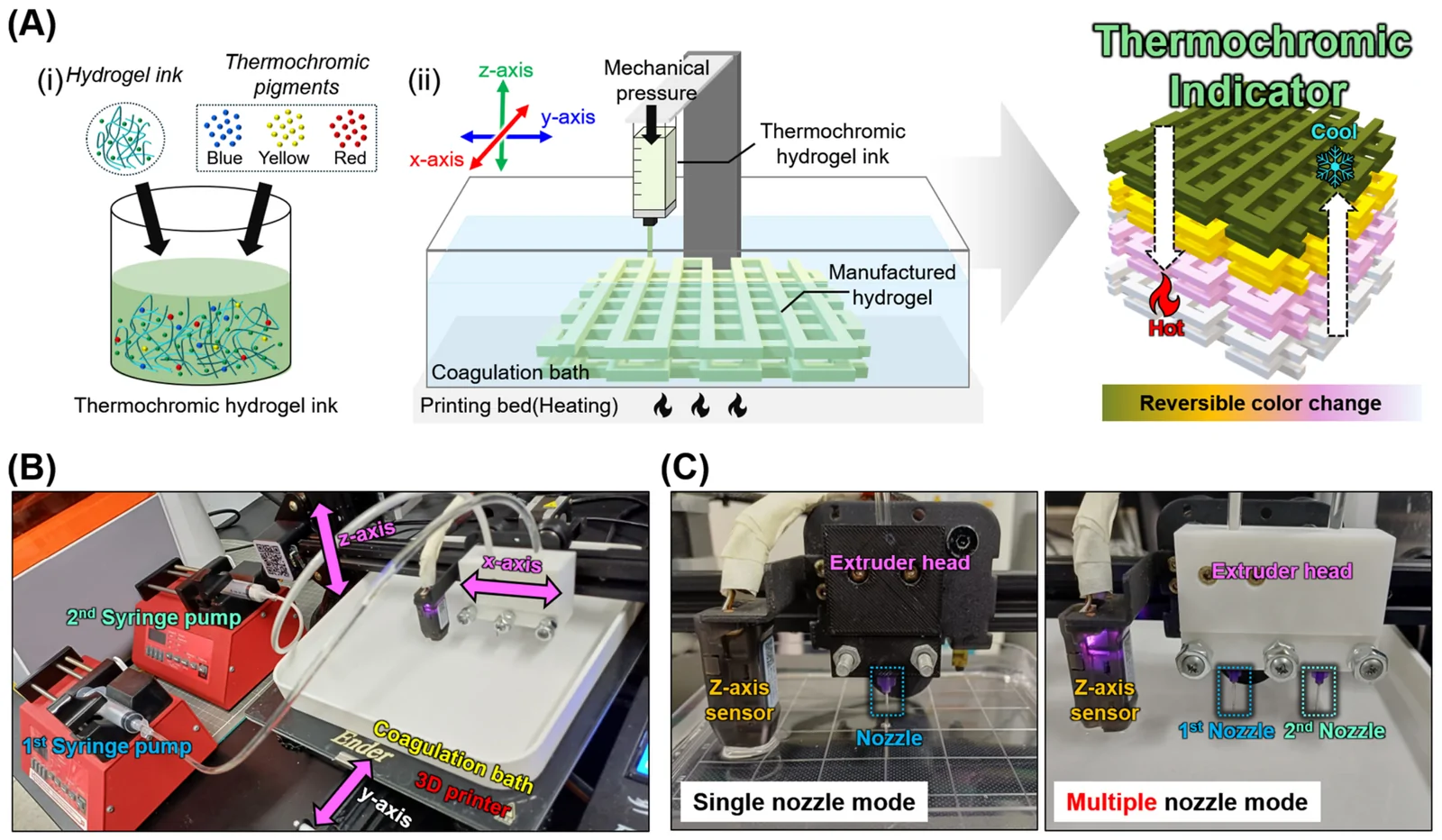
Additive-manufacturing platform and system configuration for the thermochromic hydrogel indicator. (**A**) Schematic illustration of the fabrication concept: (**i**) composition of the thermochromic hydrogel ink containing thermochromic microcapsule pigments; (**ii**) extrusion process using a heated coagulation bath and formation of multilayer constructs with reversible color transitions. Arrows indicate the reversible color change of the thermochromic indicator during heating and cooling. (**B**) Photograph of the printing setup. (**C**) Close-up view of the printhead equipped with a Z-axis sensor, single-nozzle mode (**left**) and multiple-nozzle mode (**right**).

**Figure 2:**
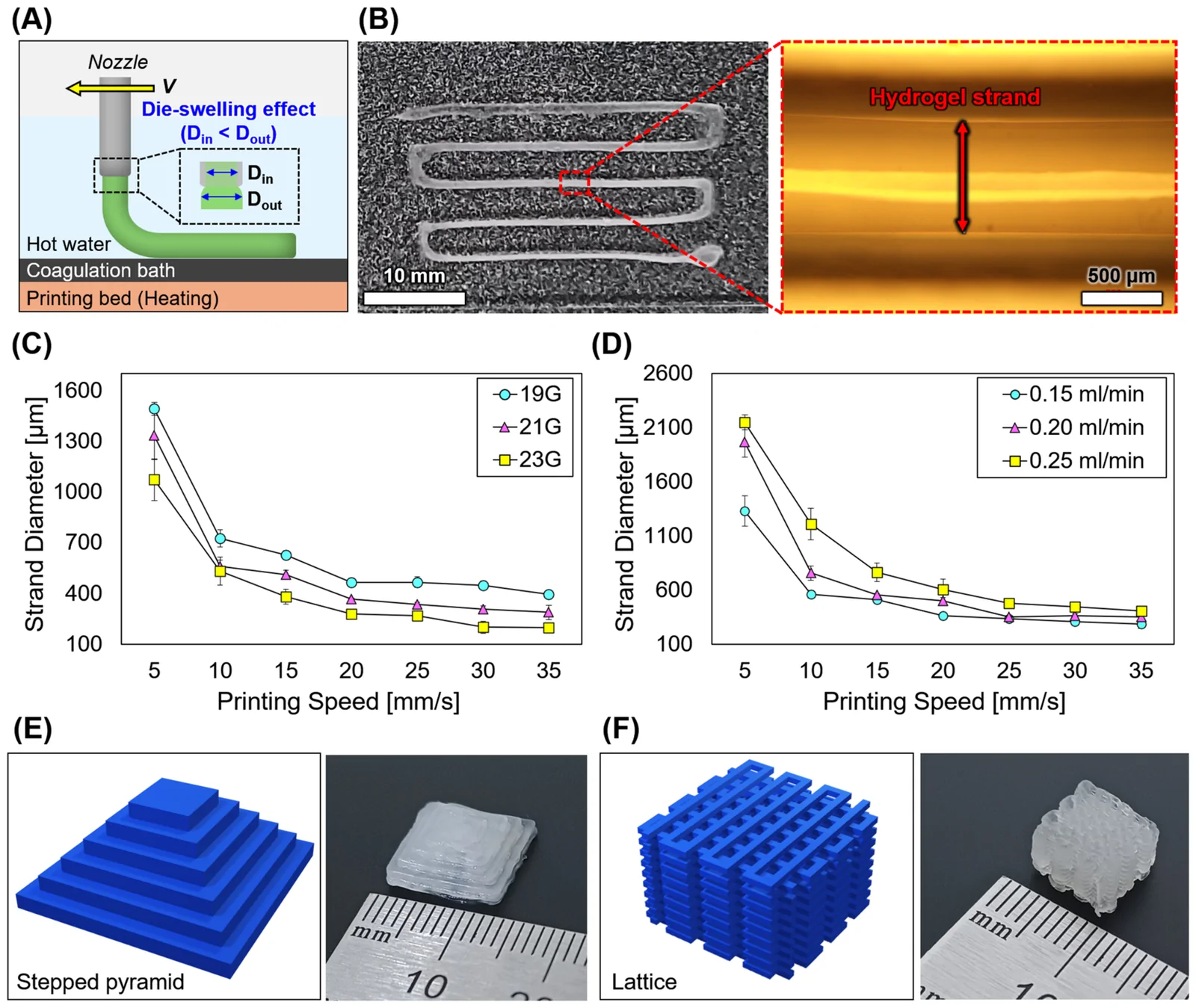
Die-swell mitigation and printing fidelity in a thermochromic chitosan hydrogel printing system. (**A**) Schematic illustration of viscoelastic die-swelling during extrusion and its mitigation using a heated coagulation bath. The arrow labeled v indicates the nozzle translation direction, while D_in_ and D_out_ denote the nozzle inner diameter and extrudate diameter, respectively. (**B**) Printed strands following the designed toolpath and magnified optical image of a single printed strand. Strand diameter as a function of printing speed for (**C**) different nozzle gauges at a 0.15 mL/min flow rate and (**D**) different flow rates using a 21G nozzle. CAD model and corresponding photograph of (**E**) a printed stepped pyramid construct and (**F**) a printed lattice construct.

**Figure 3:**
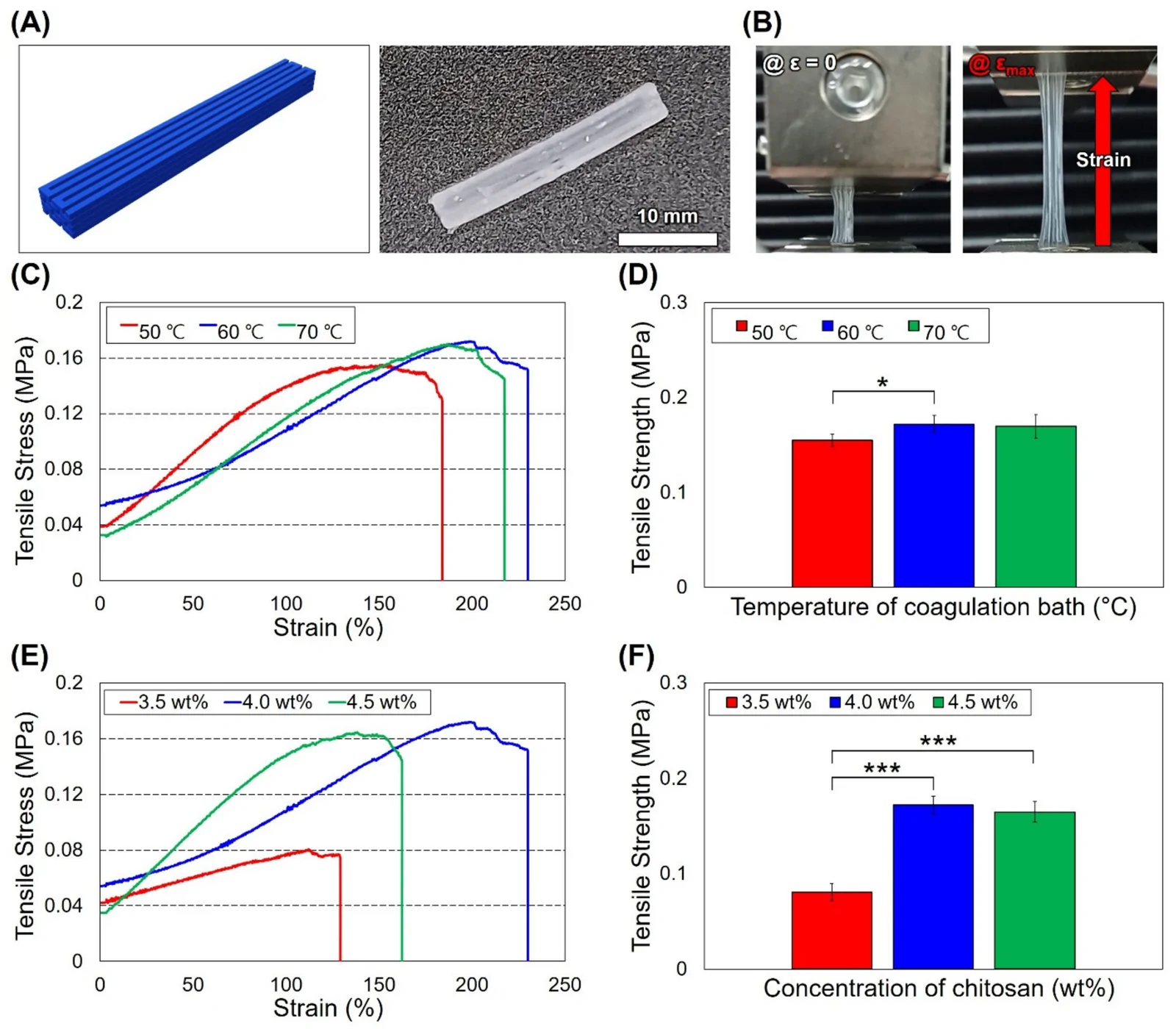
Tensile properties of chitosan hydrogel specimens as a function of coagulation bath temperature and chitosan concentration. (**A**) CAD model and corresponding photograph of a printed rectangular prism–shaped tensile specimen. (**B**) Tensile testing snapshots at ε = 0 and ε = ε_max_. The red arrow indicates the tensile loading direction. (**C**) Stress–strain curves and (**D**) comparison of ultimate tensile strength (UTS) values for specimens gelled in coagulation baths at 50, 60, and 70 °C. (**E**) Stress–strain curves and (**F**) comparison of UTS values for specimens prepared from chitosan hydrogel inks with chitosan concentrations of 3.5, 4.0, and 4.5 wt%. Statistical significance was determined using a one-way ANOVA followed by Tukey’s post hoc test. * *p* < 0.05, *** *p* < 0.001.

**Figure 4:**
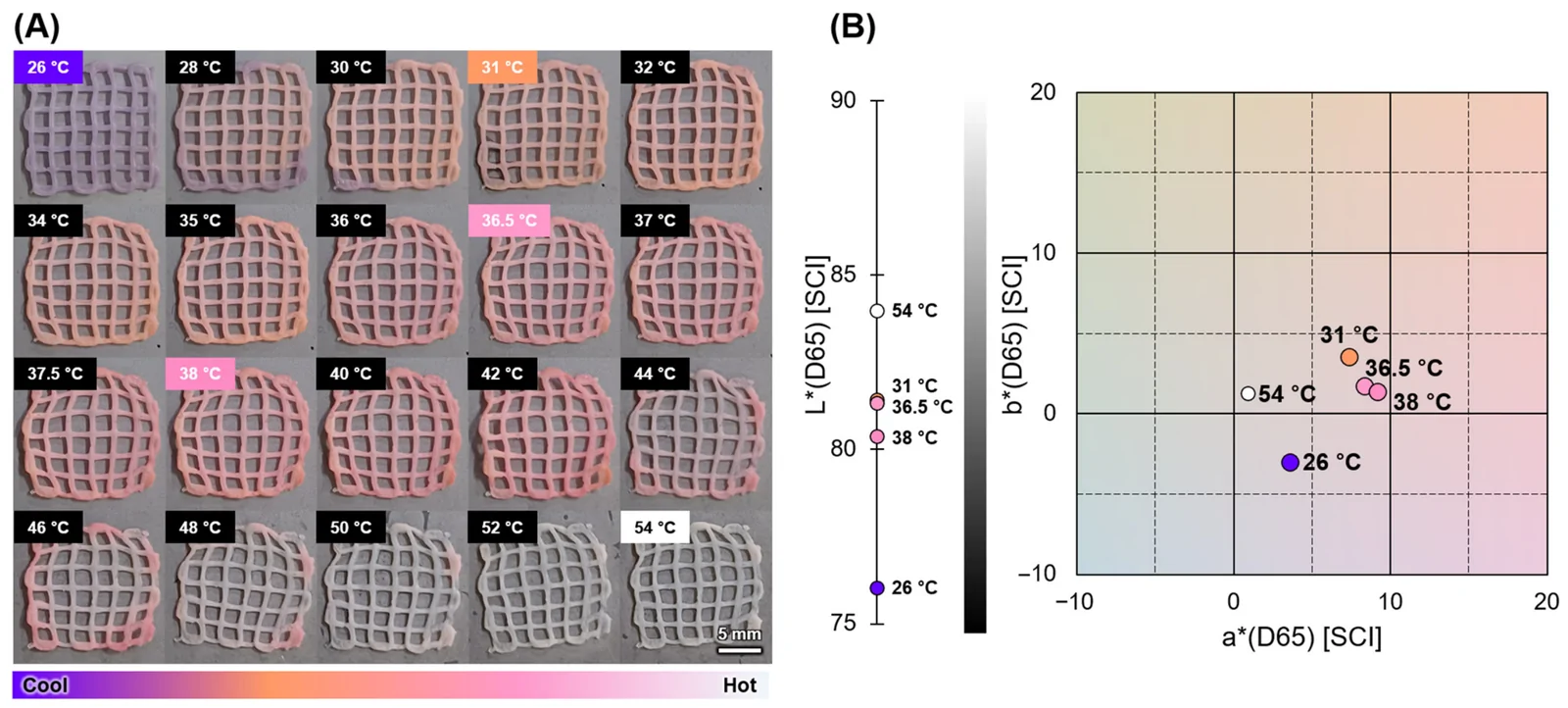
Temperature-dependent colorimetric response of a lattice-patterned thermochromic hydrogel indicator (1st formulation). (**A**) Photographs of the printed lattice indicator during heating from 26 to 54 °C, with stepwise color transitions. (**B**) Corresponding CIE L*a*b* (D65, SCI) results at representative temperatures: L* (lightness) value and a*–b* chromaticity map.

**Figure 5:**
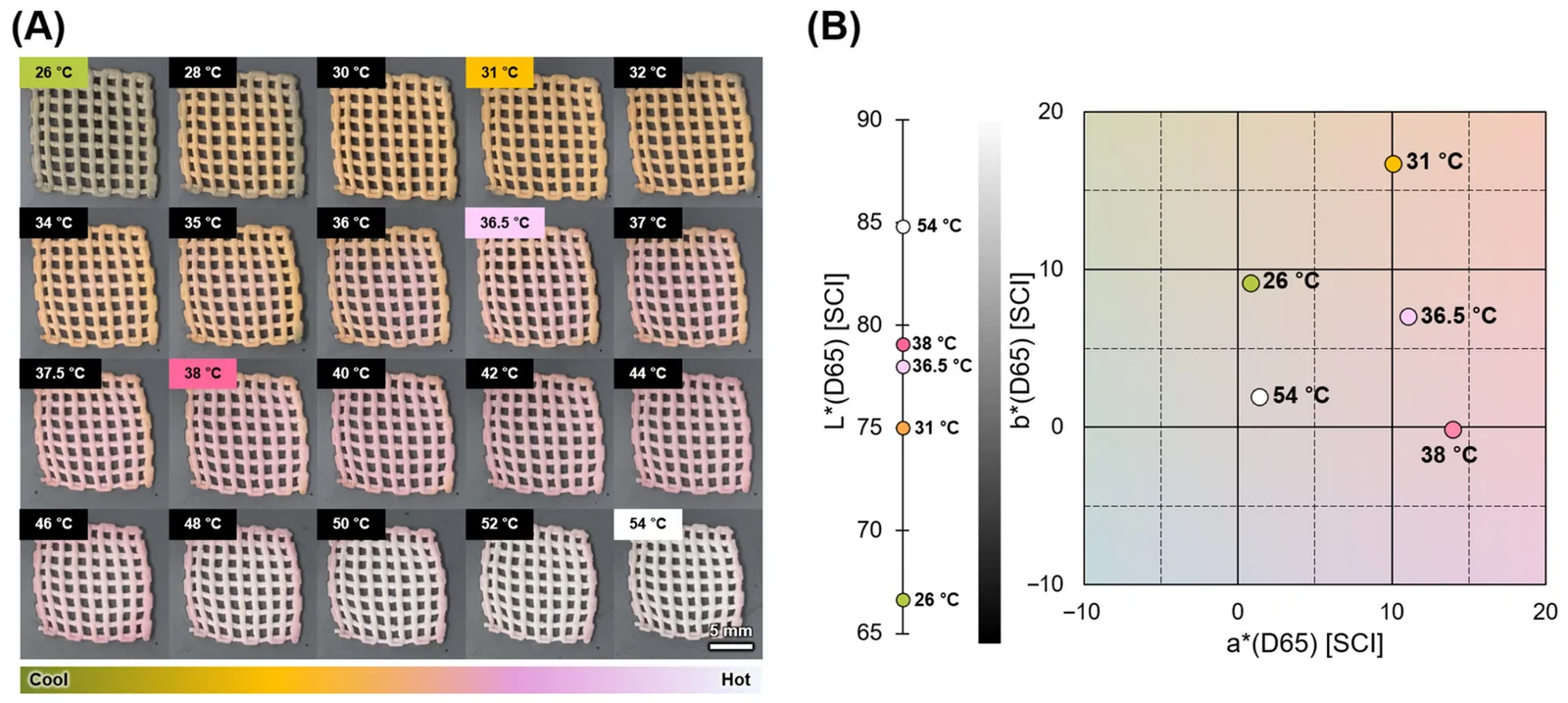
Temperature-dependent colorimetric response of a lattice-patterned thermochromic hydrogel indicator (2nd formulation). (**A**) Photographs of the printed lattice indicator during heating from 26 to 54 °C, with stepwise color transitions. (**B**) Corresponding CIE L*a*b* (D65, SCI) results at representative temperatures: L* (lightness) value and a*–b* chromaticity map.

**Figure 6:**
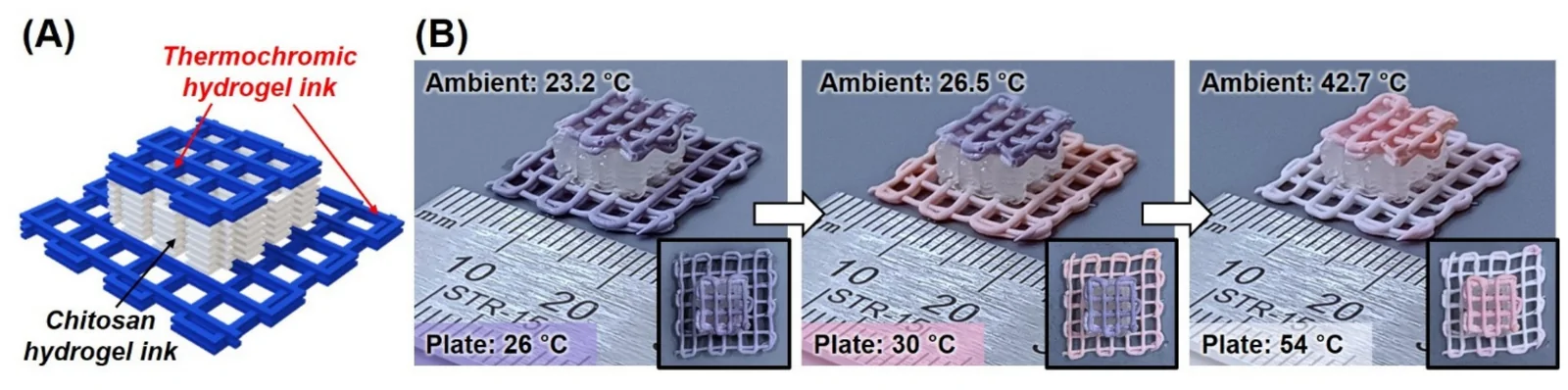
Multilayer thermochromic hydrogel indicator for layer-resolved colorimetric readout. (**A**) Schematic illustration of the stacked indicator architecture consisting of upper and lower thermochromic lattice layers separated by an intermediate chitosan hydrogel layer. (**B**) Photographs of the indicator under different ambient and plate temperature conditions. Insets: corresponding top views. White arrows indicate the progression of increasing thermal conditions, while the color changes of the thermochromic layers represent their temperature-dependent responses.

## Data Availability

The data that support the findings of this study are available from the corresponding author upon reasonable request.

## Supplementary Materials

The following supporting information can be downloaded at: https://www.mdpi.com/article/10.3390/polym18192319/s1, Figure S1: Quantitative analysis of the die-swelling ratio across different nozzle gauges (19G, 21G, and 23G) and printing speeds (5–35 mm/s). A ratio below 1.0 indicates the onset of mechanical drawing.; Figure S2. Water content and swelling ratio of printed chitosan hydrogels as a function of (A) coagulation bath temperature and (B) chitosan concentration. Bars indicate water content (left axis) and symbols indicate swelling ratio (right axis), (*n* = 5). Statistical significance was determined using a one-way ANOVA followed by Tukey’s post_hoc test.; Figure S3. Cross-sectional SEM images of chitosan hydrogels prepared at (A, A′) 3.5 wt%, (B, B′) 4.0 wt%, and (C, C′) 4.5 wt%, after oven drying.; Figure S4. pH of the immersion water for chitosan hydrogel specimens at different stages of processing (*n* = 5). The initial pH of the DI water was 7.2.; Figure S5. Mechanical stability of the thermochromic chitosan hydrogels under cyclic environmental conditions. Ultimate tensile strength (UTS) of the standardized molded hydrogel specimens (30 mm × 3.95 mm × 2.4 mm) in their initial state (Control), after 5 continuous heating (60 °C, 5 min) and cooling (room temperature, 5 min) cycles in a hydrated state (Thermal Cycling), and after a partial dehydration (60 °C oven, 1 h) and accelerated rehydration (30 °C water, 1 h) cycle (Dehydration/Rehydration). Data are presented as mean ± standard deviation (*n* = 5).; Figure S6. Color difference ($\Delta E_{ab}^{*}$) of the thermochromic indicator after 50 heating–cooling cycles and between the heating and cooling paths at the 50th cycle. The dashed line indicates $\Delta E_{ab}^{*}$ = 1, below which color differences are generally imperceptible.; Figure S7. Time-lapse images of the thermochromic hydrogel indicator after placement on a hot plate preheated to 54 °C. Elapsed time is measured from the moment of contact with the plate.; Table S1. CIE L*a*b* coordinates of the immersion water of hydrogel specimens stored for one year at 8 °C, compared with fresh DI water (mean ± SD, *n* = 5).; Table S2. Color difference ($\Delta E_{ab}^{*}$) between adjacent measured temperature states for the two thermochromic formulations (*n* = 5).; Table S3. CIE L*a*b* coordinates of the thermochromic indicator measured during heating and cooling at the 50th cycle (mean ± SD, *n* = 5).; Table S4. Color difference ($\Delta E_{ab}^{*}$) between adjacent temperature states before and after 50 heating–cooling cycles (*n* = 5).; Table S5. Measured surface temperatures of the upper and lower thermochromic layers of the multilayer indicator at each plate temperature (*n* = 5).
