# Supplementary material for: Additive Manufacturing of Thermochromic Chitosan Hydrogels for Battery-Free Colorimetric Thermal Indication
Source: Polymers (Basel). 2026 Sep 22;18(19):2319. doi: 10.3390/polym18192319 (PMC13666924; doi:10.3390/polym18192319)
Supplement: Supplementary file 1 [file polymers-18-02319-s001.zip › polymers-4540034-supplementary.pdf]

Supporting Information

Additive Manufacturing of Thermochromic Chitosan Hydrogels for Battery-Free Colorimetric Thermal Indication

Jaewon Lim [1,†], Na Kyoung Kim [1,†], Jong Woo Hah [1], Yoo Sei Park [2,3], Inhwan Lee [1], Hyoryung Nam [4,*], Geon Hwee Kim [1,*]

[1]Department of Mechanical Engineering, Chungbuk National University (CBNU), 1, Chungdae-ro, Seowon-gu, Cheongju-si, Chungcheongbuk-do, 28644, Republic of Korea

[2]Department of Nanoenergy Engineering, Pusan National University, Busandaehak-ro 63 beon-gil 2, Geumjeong-gu, Busan, 46241, Republic of Korea

[3]Department of Nano Fusion Technology, Pusan National University, Busandaehak-ro 63 beon-gil 2, Geumjeong-gu, Busan, 46241, Republic of Korea

[4]Department of Biomedical Engineering, Daegu Catholic University School of Medicine, 33 Duryugongwon-ro 17-gil, Nam-gu, Daegu, 42472, Republic of Korea

[*]Co-corresponding authors: ryung@cu.ac.kr (H.N.); geonhwee.kim@chungbuk.ac.kr (G.H.K.)

[†]These authors contributed equally to this work.

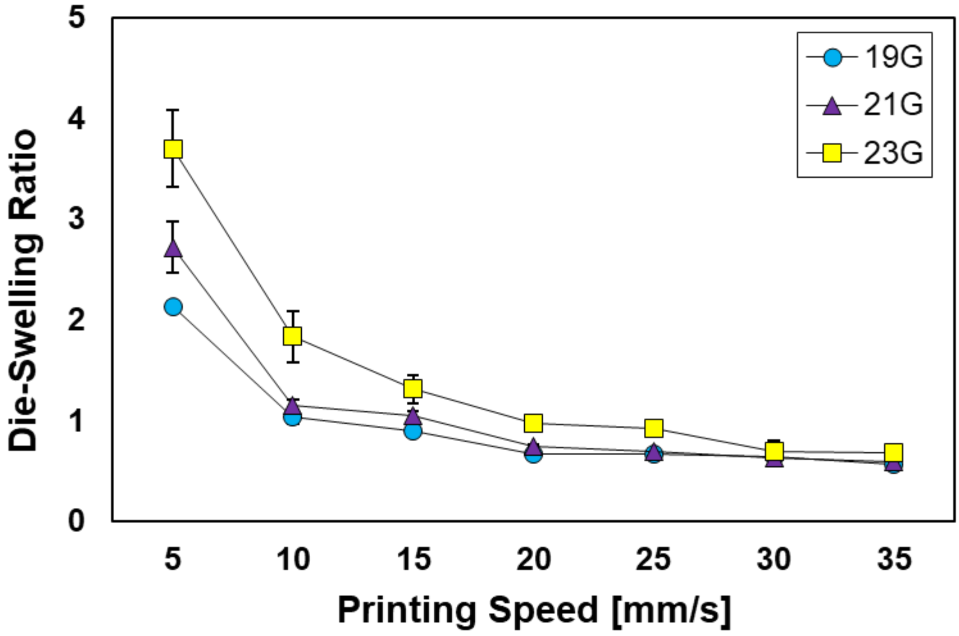

Figure S1. Quantitative analysis of the die-swelling ratio across different nozzle gauges (19G, 21G, and 23G) and printing speeds (5–35 mm/s). A ratio below 1.0 indicates the onset of mechanical drawing.

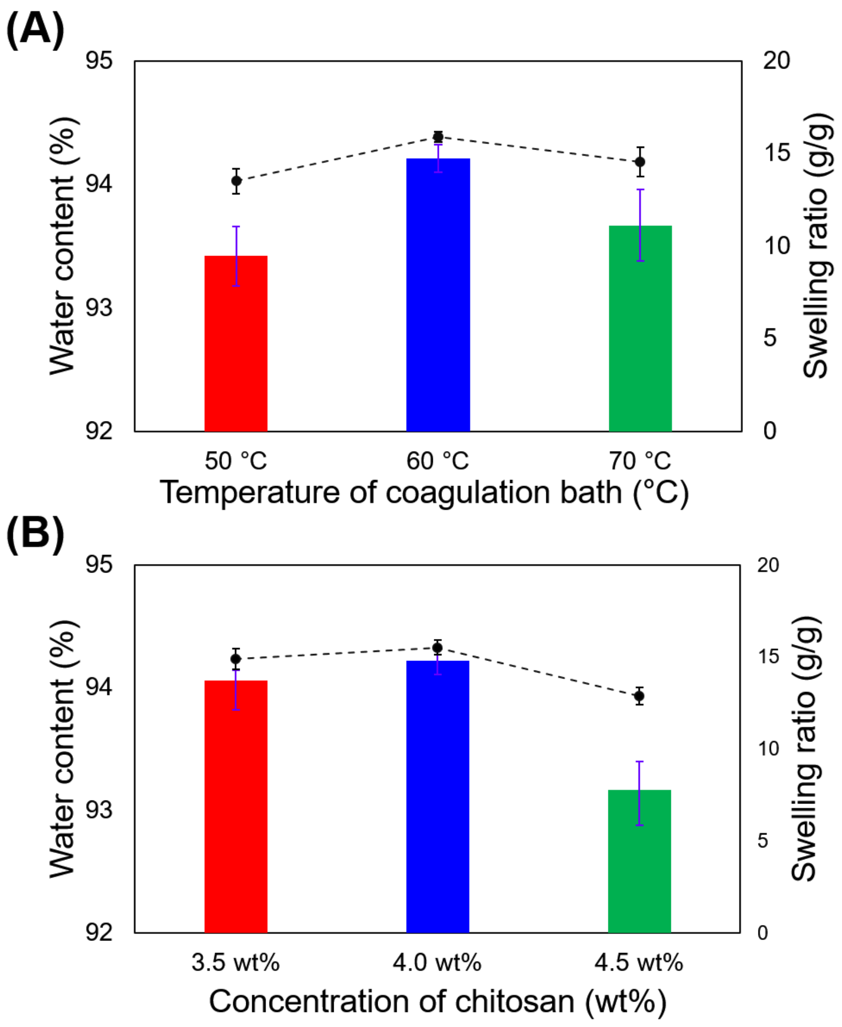

Figure S2. Water content and swelling ratio of printed chitosan hydrogels as a function of (A) coagulation bath temperature and (B) chitosan concentration. Bars indicate water content (left axis) and symbols indicate swelling ratio (right axis), (*n* = 5). Statistical significance was determined using a one-way ANOVA followed by Tukey's post_hoc test.

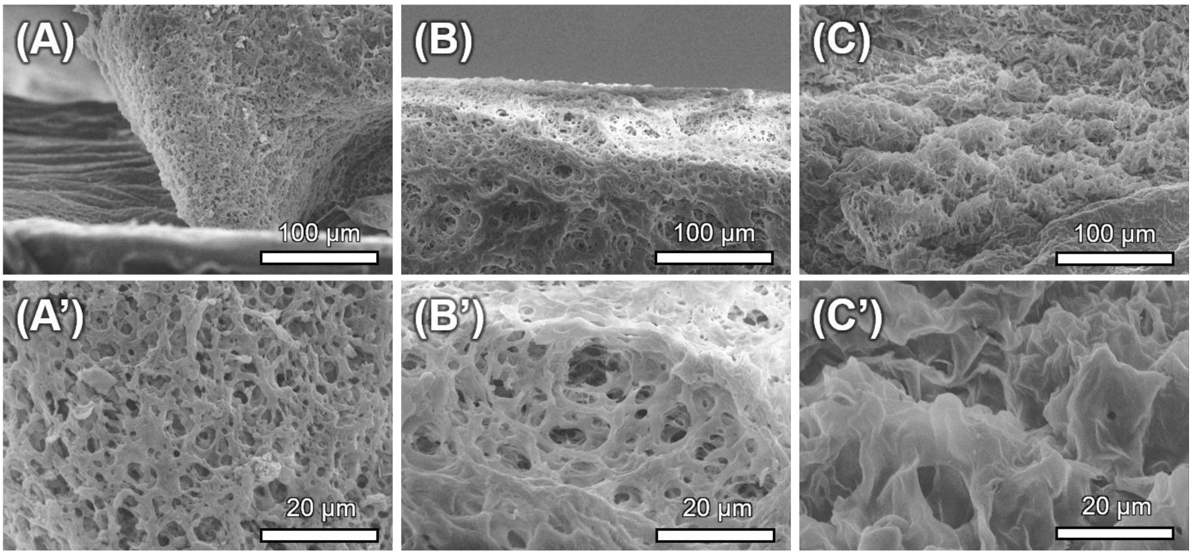

Figure S3. Cross-sectional SEM images of chitosan hydrogels prepared at (A, A′) 3.5 wt%, (B, B′) 4.0 wt%, and (C, C′) 4.5 wt%, after oven drying.

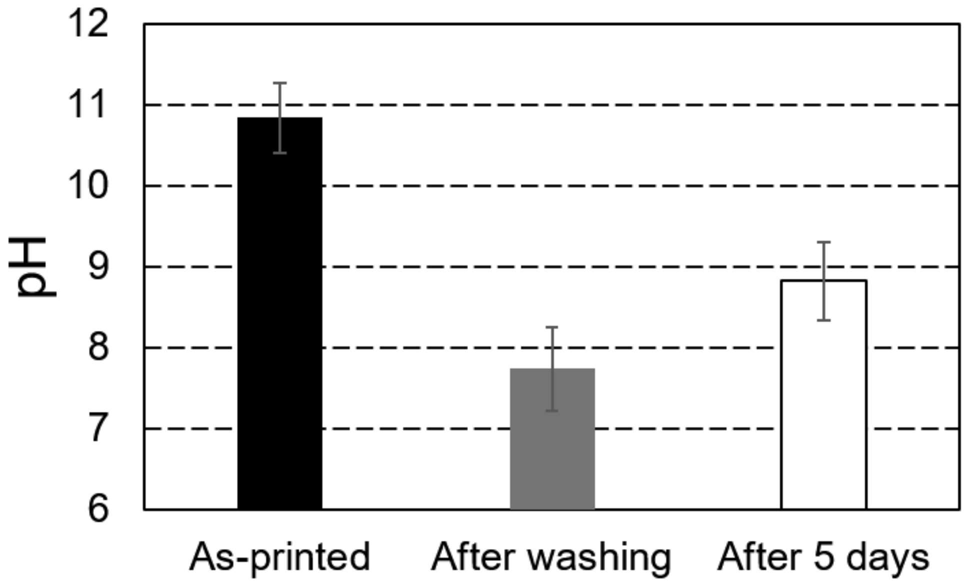

Figure S4. pH of the immersion water for chitosan hydrogel specimens at different stages of processing (*n* = 5). The initial pH of the DI water was 7.2.

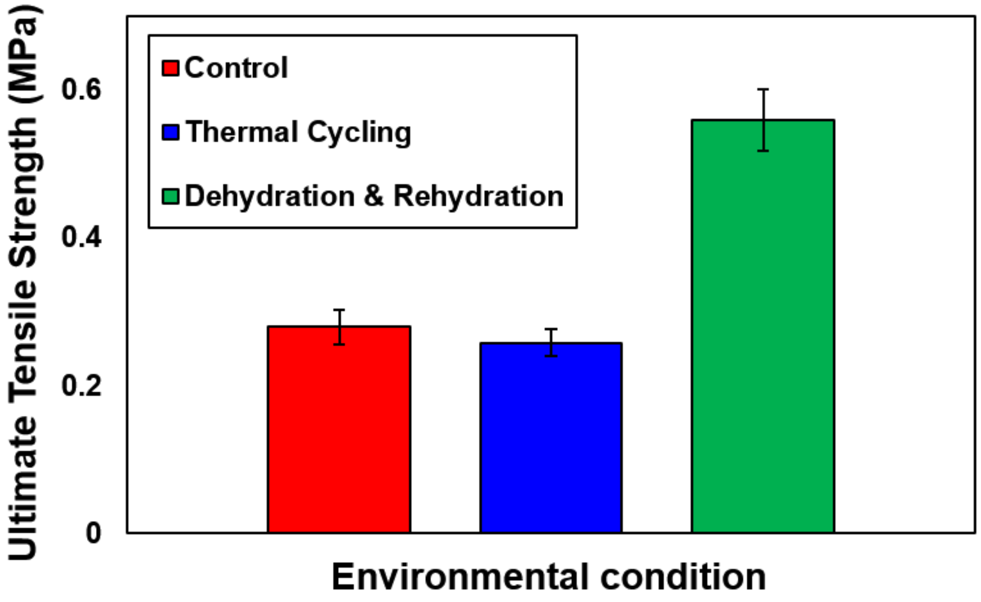

Figure S5. Mechanical stability of the thermochromic chitosan hydrogels under cyclic environmental conditions. Ultimate tensile strength (UTS) of the standardized molded hydrogel specimens (30 mm × 3.95 mm × 2.4 mm) in their initial state (Control), after 5 continuous heating (60 °C, 5 min) and cooling (room temperature, 5 min) cycles in a hydrated state (Thermal Cycling), and after a partial dehydration (60 °C oven, 1 h) and accelerated rehydration (30 °C water, 1 h) cycle (Dehydration / Rehydration). Data are presented as mean ± standard deviation (*n* = 5).

	L*(D65)	a*(D65)	b*(D65)
Fresh DI water	45.806±1.74	0.28±0.02	-1.306±0.07
Immersion water	46.028±1.68	0.27±0.02	-1.086±0.1

Table S1. CIE L*a*b* coordinates of the immersion water of hydrogel specimens stored for one year at 8 °C, compared with fresh DI water (mean ± SD, *n* = 5).

Transition	1st formulation	2nd formulation
26 → 31 °C	9.35 ± 0.82	14.63 ± 0.28
31 → 36.5 °C	2.59 ± 0.47	10.19 ± 0.28
36.5 → 38 °C	1.57 ± 1.26	7.80 ± 0.34
38 → 54 °C	9.06 ± 1.24	13.96 ± 0.52

Table S2. Color difference (ΔE^*_{ab}) between adjacent measured temperature states for the two thermochromic formulations (*n* = 5).

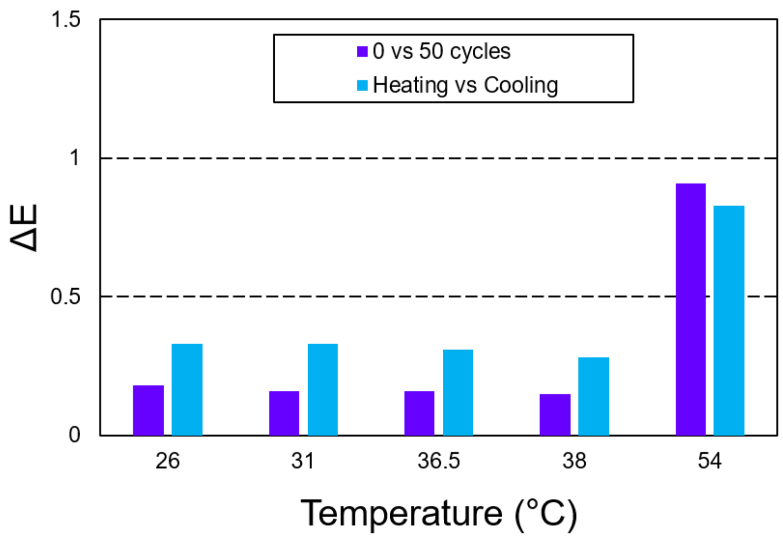

Figure S6. Color difference (ΔE^*_{ab}) of the thermochromic indicator after 50 heating–cooling cycles and between the heating and cooling paths at the 50th cycle. The dashed line indicates ΔE^*_{ab} = 1, below which color differences are generally imperceptible.

Temperature (°C)	Heating: L*(D65)	a*(D65)	b*(D65)	Cooling: L*(D65)	a*	b*
26	76.18 ± 1.49	3.55 ± 0.39	−2.95 ± 0.83	75.91 ± 1.39	3.67 ± 0.24	−3.10 ± 0.56
31	81.30 ± 0.77	7.47 ± 0.51	3.49 ± 0.35	81.56 ± 0.32	7.31 ± 0.16	3.62 ± 0.16
36.5	81.45 ± 1.26	8.27 ± 1.17	1.79 ± 0.27	81.21 ± 0.30	8.43 ± 0.37	1.68 ± 0.16
38	80.49 ± 0.86	9.08 ± 0.96	1.42 ± 0.47	80.28 ± 0.25	9.24 ± 0.19	1.33 ± 0.40
54	84.88 ± 0.43	0.94 ± 0.25	1.31 ± 0.18	84.06 ± 0.81	0.87 ± 0.21	1.25 ± 0.15

Table S3. CIE L*a*b* coordinates of the thermochromic indicator measured during heating and cooling at the 50th cycle (mean ± SD, *n* = 5).

Transition	0 cycle	50 cycles
26 → 31 °C	9.35 ± 0.82	9.11 ± 1.17
31 → 36.5 °C	2.59 ± 0.47	1.88 ± 0.68
36.5 → 38 °C	1.57 ± 1.26	1.31 ± 1.47
38 → 54 °C	9.06 ± 1.24	9.25 ± 0.99

Table S4. Color difference (ΔE^{*}_{ab}) between adjacent temperature states before and after 50 heating–cooling cycles (*n* = 5).

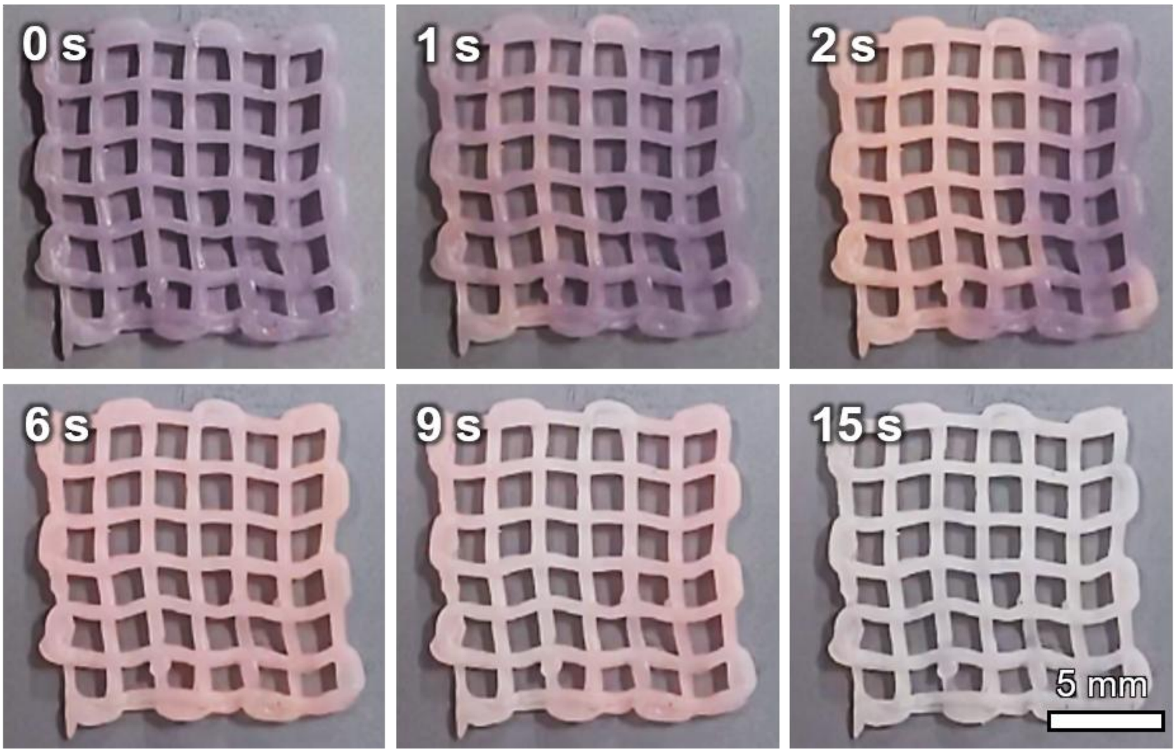

Figure S7. Time-lapse images of the thermochromic hydrogel indicator after placement on a hot plate preheated to 54 °C. Elapsed time is measured from the moment of contact with the plate.

Plate temperature (°C)	Upper layer (°C)	Lower layer (°C)	ΔT (°C)
26	23.6 ± 0.8	25.2 ± 0.8	1.6
30	26.4 ± 0.9	29.1 ± 1.0	2.7
54	43.7 ± 0.7	52.6 ± 1.6	8.9

Table S5. Measured surface temperatures of the upper and lower thermochromic layers of the multilayer indicator at each plate temperature (*n* = 5).
